# Approaches to integrating online videos into health professions curricula: educators’ perspectives from multiple institutions

**DOI:** 10.12688/mep.19179.1

**Published:** 2022-07-12

**Authors:** Kelly Aluri, Mohamed Sow, Manuel Amieva, Sharon Chen

**Affiliations:** 1 Stanford University School of Medicine, Stanford, CA, 94304, USA

**Keywords:** medical education research, microbiology, educational environment, curriculum development, teaching & learning

## Abstract

**Background: **The COVID-19 pandemic has accelerated a transition from lecture-based classes to blended and online learning, increasing the need to integrate publicly available online educational videos. Although online videos are widely available, it is challenging for educators to effectively integrate them into a curriculum. Years before the pandemic, educators from different institutions integrated videos from a library of microbiology and immunology resources into different curricula. Their experiences may inform current educators on the approach to incorporating external resources into their unique curricula.

**Methods:** We interviewed US health professions instructors or course directors who had previously requested access to online microbiology and immunology videos. Using thematic analysis, we organized prominent themes into an existing framework for curriculum development. We then reflected on the meaning of the themes using the same conceptual framework.

**Results:** We found that educators from different schools were able to integrate the same publicly available videos into varying contexts. Most used them as preparation for interactive sessions. For integrating videos, educators felt success when the following actions occurred. 1) Educators integrated videos as a tool to enhance active-learning activities. 2) Educators created activities that focused on clinical applications of knowledge, taught critical thinking, and developed enthusiasm for the subject. 3) They tested students on knowledge application and major concepts rather than solely on content for high-stakes exams. 4) Educators worked with administrators who understood the goals of integrating external videos and supported educators with time and resources to develop effective blended learning.

**Conclusion:** Our study suggests that educators integrating external resources into their curricula may benefit from first establishing their goals and aspirations for their students. These goals then become the anchor for other curricular elements, including external videos, in-class activities, and assessments. Our study highlights the need for dedicated time to develop experienced and enthusiastic educators.

## Introduction

The COVID-19 pandemic has accelerated a global transition from lecture-based classrooms to blended and online learning with a high demand for readily available online educational videos in the public domain ( Gordon *et al.*, 2020). Many educators rapidly adopted blended learning, mixing asynchronous online videos with synchronous real-time virtual teaching ( Consorti *et al.*, 2021; Dost *et al.*, 2020; Motte-Signoret *et al.*, 2021). Although online medical education videos are widely available, it can be daunting for educators to determine how to best use online videos and when to customize their own ( de Jong *et al.*, 2020; Dong & Goh, 2015). During the COVID-19 pandemic, instructors often hastily applied reactionary changes to sustain instruction for their students. The rapid pivot towards blended learning has been associated with student dissatisfaction, reflecting a potential lack of experience for effectively integrating online learning tools ( Mohammed, 2020; Mortazavi *et al.*, 2021). Medical school instructors that pivoted teaching methods during the pandemic reported a need for structures that support online learning, as well as best practices underpinned by theories ( Daniel *et al.*, 2021; Gordon *et al.*, 2020). With more time for deliberate planning, educators can now reassess and potentially retain learning adjustments they were forced to make.

To extract lessons to inform the post-pandemic medical education environment, we evaluated how educators from different institutions used an identical set of online medical education videos. Years before the COVID-19 pandemic, a group of medical educators from 4 institutions created a comprehensive library of Microbiology and Immunology (M&I) videos ( Adam *et al.*, 2017; Chen *et al.*, 2019). Other educators, who were not part of the project, specifically accessed these online videos. They individually planned integration of the online videos into their courses without specific instructions from the video creators. We believed that these engaged educators’ experience of integrating online videos into their courses replicates the situation and challenges of current medical school educators, who have also had to individually plan integration of online videos from external sources. 

With this unique scenario, we aimed to capture how 10 programs approached integrating this specific set of online videos into very different courses. We conducted a qualitative study to gain a comprehensive view of the educators’ thought-processes, successes, and challenges. We used a theoretical framework for health professions curriculum development as a lens to view the participants’ insights ( Lee *et al.*, 2013). Lee *et al.*,’s framework for curriculum development emphasizes the dynamic, integrated nature of curriculum ( Lee *et al.*, 2013). While much of the current literature addresses student satisfaction with and reactions to online learning, our study addresses an area of limited study—instructors’ cognitive processes and behaviors when integrating external learning resources into their curriculum ( Stojan *et al.*, 2022).

## Methods

### Participants and recruitment

We selected participants from educators who requested access to our open-source M&I videos and formally agreed to be contacted in the future. We emailed the educators directly to ask about participation in our study. We interviewed ten participants who were instructors or course directors of a microbiology and/or immunology course at an accredited U.S. medical or osteopathic school.

### Data collection

We interviewed participants via video conferencing. One investigator (KZA) conducted all interviews, which lasted 30 to 45 minutes each. To promote confidentiality and open reflection, both interviewer and participants were located in private spaces. With the participant’s informed consent, we audio recorded the interviews. Consent was obtained verbally and recorded at the beginning each interview. We removed personal or institutional identifying information from the recordings. We transcribed the recordings using a secure, automated transcription service, and then we reviewed for accuracy. The Institutional Review Board at Stanford University approved our study.

The interviews were semi-structured, with open-ended questions in three main domains: 1) how instructors integrated M&I videos into their curriculum, 2) how instructors used videos to achieve their goals, and 3) how instructors assessed student learning. We asked participants to describe elements of their curriculum, the student experience of the course, the benefits and challenges of using online videos for the course, and institutional or instructor factors that contributed to the success of the course. Interview questions were informed by the authors’ research questions on the different experiences with integration of online videos, current literature on integration of external resources into curricula, and Lee *et al.*,’s framework for curriculum development in health professional education ( Lee *et al.*, 2013). The interview guide was piloted with three instructors at the authors’ home institution.

### Data analysis

We analyzed the data with NVivo 12.0, using thematic analysis, a methodology that uses a six-step process to generate themes ( Kiger & Varpio, 2020). While Nvivo is a proprietary software, the same analysis can be conducted using open source software such as FreeQDA and QDA Miner Lite. Two investigators (KZA and MS) coded every interview to maximize internal validity and coding consistency. First, analysts gained familiarity with the data by reviewing the transcripts. Initial codes were generated, and then codes were organized into potential overarching themes. Themes that persisted across interviews or seemed impactful to integrating online videos were defined as “key”. The key themes were reviewed and refined until team consensus was reached. Exemplar quotations from the interviews were associated with the key themes. We reflected on the meaning of the key themes based on a conceptual framework for curriculum development ( Lee *et al.*, 2013). 

## Results

The 10 interviewed educators taught at U.S. institutions in 9 different states. Their relevant characteristics, including educational background, role in their current institution’s program, and the type of program, are listed in Table 1.

**Table 1.** Characteristics of study cohort.

Characteristic	Number (n =10)
**Academic Training** MD PhD MD, PhD	3 6 1
**Length of Employment in Program** Mean (SD)	6.2 (3.3)
**Role in Program** Course director Professor or lecturer Associate dean	5 3 2
**Type of Program** MD DO PA	8 1 1
**Students in Course** MS1/PA1 MS2 MS3 MS1 and MS2	4 3 1 2
**Gender** Female Male	6 4

To understand each school’s process of integrating online videos into their curriculum, we first characterized their distinct education environments. While most educators worked for medical schools, one was a course director at an osteopathic medical school and one was a course director in a physician assistant training program. The educators led courses ranging from introductory immunology for first year students to a multidisciplinary microbiology elective for clinical students.

Six educators described integrating M&I videos during a time of school-wide curriculum changes, which compelled them to use external M&I videos. Some of the curriculum changes shortened time for preclinical teaching, leading educators to provide content outside of class. Other schools transitioned from the lecture-based model to flipped classroom style instruction. Eight schools envisioned using the videos to provide context for in-class case studies.

### Themes

**Figure 1.**
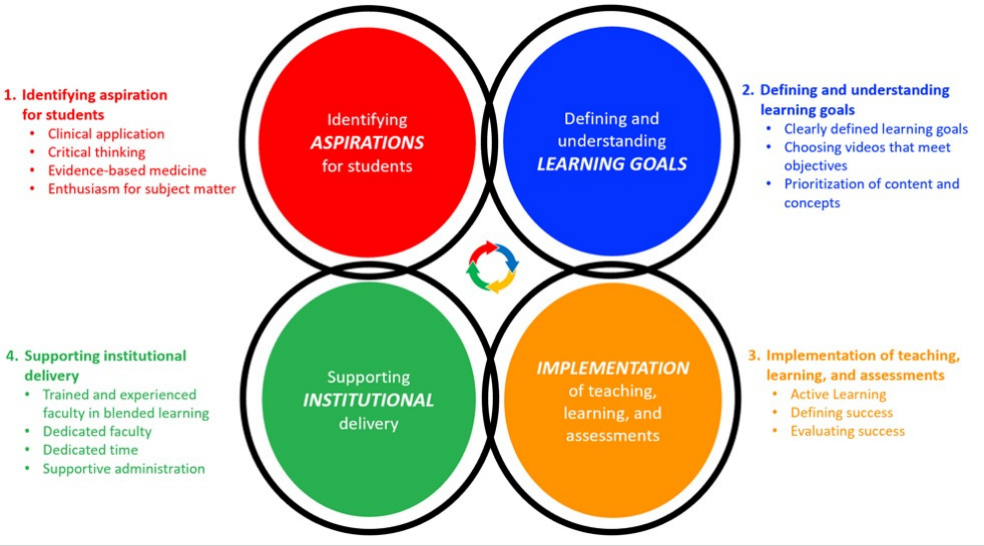
Conceptual framework for curriculum development with publicly available videos and resources.

We organized the key themes around a conceptual framework for curriculum development ( Lee *et al.*, 2013). This framework emphasizes the inter-related nature of curricular elements (dimensions), which allows us to explore their connections. In the original framework, the inter-related dimensions are: 1. Identifying future healthcare practice needs, 2. Defining and understanding capabilities (that health professions require), 3. Teaching, learning, and assessment, 4. Supporting institutional delivery. We present our key themes using the framework, adapting the descriptors of each dimension ( Figure 1).

Our analysis revealed that educators who had a positive outlook and perceived success about integrating external videos thought about the connections between the curricular elements and viewed external videos as a tool, not a centerpiece. For example, when asked about their course’s goals, educators naturally described the type of healthcare provider they wanted their students to be (Dimension 1). Some educators were able to set learning objectives consistent with their aspirations for students (Dimension 2). Specifically, educators who valued critical thinking prioritized teaching about concepts and applications over rote memorization. However, some educators ran into heavy institutional and student pressure to teach specific content (Dimension 4), resulting in an inconsistency between curriculum dimensions.

### Identifying aspirations for students

We described Dimension 1 of the framework as: “ *Identifying educator’s aspirations for students*”. Educators expressed that their goal for their students was to become adept clinicians. They identified qualities clinicians needed, such as applying knowledge, thinking critically, using evidence-based medicine, and maintaining enthusiasm for medicine. They felt that transferring basic content through external videos before class helped them generate initial interest in the subject matter and allot more time for activities that teach higher level skills.

*“When you're in class, you’re supposed to be using your brain. It's not about sitting back and trying to absorb passively. We're in clinical training programs, so let's think like clinicians, let's use our brains.” (10)*
*“[I’m] a fan of the patient springboard videos that I just feel like they do a nice job of clinical relevance… they intertwine immunology, microbiology, infectious disease, but it's in the context of a patient.” (1)*
*“We're trying to emphasize that medicine is evidence-based, but also sometimes you have incomplete pieces of data... In fact, you probably will never have all the answers. But I'm sure you still have to make decisions.” (1)*
*“I want them to get excited about this course... generating that enthusiasm is really important.” (9)*

### Defining learning and understanding learning goals

*We described Dimension 2 as “Defining and understanding learning goals”.* Educators who expressed a positive outlook on integrating external videos created goals that aligned with the aspirations for students. For example, educators who prioritized critical thinking set their learning goals to achieve this attribute. They focused on curating videos that would allow more class time for clinical application and critical thinking.

*“I want people to be able to engage with the next organism and respond the way some of the leaders that we've witnessed have responded to this pandemic, to understand the complexity of the biology and come up with strategies to mitigate.” (6)*
*“You definitely have to curate the ones that are relevant. I made a decision that I was going to watch every single video that would possibly be relevant to my goals.” (1)*
*“These videos are not your entire curriculum. Just because you have this content doesn't mean you have a course. The videos are great. That's the basic knowledge that someone needs to know. [But] you need creativity for the interactive sessions to format it into a way that's going to work for you and your course. Even if you have all the materials, you still have to put work in there.” (10)*

Some educators reported challenges in balancing delivery of detailed content (i.e. lists of facts) versus broader concepts through critical thinking. They felt pressure from students and administrators to prioritize teaching of detailed content for high-stakes exam (i.e USMLE, US Medical Licensing Examination) preparation. The educators never identified good test-takers or expert memorizers as their aspiration for students, creating an inconsistency between Dimension 1 and 2.

*“It makes it very difficult because there's a lot of material. It's sometimes difficult to separate in your mind what's basic and should already be covered in the video. And what do I really need to talk about in this interactive part? And so it's, it's actually tough to create.” (10)*
*“There were many [barriers to implementation]. There was skepticism about [implementing the flipped classroom model and using external videos] from the administration. [They] think that teaching is about delivery of content and prize that over [taking] time to help people think.” (4)*

### Integration of teaching, learning and assessment

We described Dimension 3 as *“Integration of teaching, learning, and assessment*.” To meet their learning goals in Dimension 2, many of the educators used some active learning strategy in their course, including group discussions, polling, and interactive case studies. For assessment of their teaching, some identified exam achievement, but many educators defined student success by referring to their original aspirations for their students (Dimension 1). Success was defined as a positive change in attitude towards the subject, better critical thinking skills, and/or the ability to thrive in a clinical setting.

*“[During] those clinical experiences, [success is when] they feel like the class has made them feel more confident in the clinical setting. The information they're getting will make them walk into that clinical experience and be like, ‘Oh, I don't feel totally out of place in a clinical immunology setting.’” (1)*
*“In the long-term, beyond boards… in the clinical setting, really feel comfortable with the micro. Be able to just answer those questions on the spot at the bedside when their preceptor is asking them something.” (4)*

Although many educators identified student success based on original aspirations, their evaluation tools were limited to testing short-term retention of knowledge from external videos and rarely measured their definitions of student success, including attitude changes, critical thinking skills or confidence in a clinical setting. The inconsistency between educators’ goals (Dimension 1) and assessment tools (Dimension 3) created an inconsistency between the elements of the curriculum development framework, which renders it challenging to assess whether the use of external videos helped achieve instructors’ original goals.

### Supporting institutional delivery

*We described dimension 4 as “Supporting institutional delivery”.* Over half of educators described that dedicated faculty, protected time, and a supportive administration were all required to integrate online videos effectively.

Educators who expressed a positive outlook on integrating online videos worked with an administration who supported the consistency between aspirations for students (Dimension 1), course learning goals (Dimension 2), and execution of class time (Dimension 3). Inconsistencies between the dimensions led to both educators and administrators to view online videos unfavorably. For example, while an instructor may aspire to teach students clinically relevant thinking, an institution’s goal may be to transfer facts and utilize the knowledge of their faculty.

*“[Some administrators and faculty] were very against the idea that we would use another institution’s learning materials. That was felt to be an embarrassment. [They] felt… our students are paying for our knowledge, not the knowledge of somebody else in the videos.” (4)*
*“If we have to edit someone else's materials, we might as well produce them ourselves…We occasionally have used outside materials for learning, but mostly we've generated our own or we'll [have] live lectures. We actually have our own internal instructional design team. So we are in the process of generating more non-live, asynchronous learning materials.” (8)*

Despite administrative support, educators tallied several key “needs” that could be challenging to find. The “right” individuals with experience and enthusiasm was a key need that educators pointed out.

*“I think that [faculty] need to experience [flipped classroom] themselves. They have to experience the type of teaching almost as a student before you can really understand what is possible.” (4)*
*“Not all faculty members had ever experienced anything like that, let alone know how to design a session like that…It's a huge job to do... Most faculty members still are not familiar with those kinds of teaching techniques.” (5)*
*You need people who are going to own this. If you just tell some random person I need you to do X and they're not really excited about it, it's not going to happen...Even if there's content that's written for you…you still have to have people to implement it.” (10)*
*“Without having faculty buy-in, it's not going to happen. And that's something that we find all medical schools are like this. If you don't have a champion, it doesn't really happen or it doesn't happen well.” (8)*

Thoughtful curriculum design also means offering dedicated or protected time to enable success and to engage with a community for learning. Both of these “needs” were hard to find.

*“So the challenge is just having the time, you know… how much time do I have to watch 40 hours of material on top of my day job. It's just time.” (1)*
*“Time. Honestly, that's really what it is. You need time to do it.” (10)*
*“Yes, [knowing what other schools are doing] would be awesome. Because more brains means better integration of these videos.” (9)*

Instructors especially need time during the first iteration of the course and its transition from more traditional teaching models to new flipped classroom models.

*"The clinicians who are mostly the teachers in the course are so short on time...You can have the best intentions [to improve your course, but] when it gets down to it and you're supposed to do discussion in two days, you just go back to what you used to do.” (4)*

## Discussion

We interviewed engaged health professions educators from multiple learning environments who integrated freely available microbiology & immunology videos into their courses. Their experiences may be helpful to current educators who are optimizing for the post-pandemic learning environment. Grounding our analysis in an existing framework on curriculum development, we found that successful integration of online videos is promoted by certain behaviors of the educator. For example, educators who were mindful of the goals and aspirations for their students viewed integrating online videos more favorably. We focused our study on instructors’ thinking and behaviors as course directors and as a member of a larger organization, because these perspectives are uncommon in current published literature about the use of technology tools ( Stojan *et al.*, 2022).

After reflecting on the insights of the educators we interviewed, we generated 2 key takeaways. The first takeaway is for educators to deliberate on curriculum design as interrelated dimensions of a framework that dynamically affect each other, rather than a linear process ( Figure 1). Paying attention to the consistency between dimensions leads educators to understand that videos are a tool to achieve their objectives, rather than a centerpiece of the course. Educators perceived success about integrating videos if they set their learning objectives and assessments to be consistent with their aspirations for their students. In contrast, inconsistencies between these dimensions led to educators to have more frustrating experiences with online video integration. For example, when assessments emphasized facts rather than concepts, the content of the videos can become the center of attention, leading administrators to be resistant to using outside resources and to encourage educators to make their own.

Our second takeaway is for administrators to provide time and training to improve blended learning. The process of curriculum development described in the framework requires time for instructor training, curriculum development, and thoughtful reiteration ( Motte-Signoret *et al.*, 2021). The educators encountered time and resource constraints, which sometimes prevented integration of the videos and execution of their vision for the course. Without time and skills, instructors are more inclined to revert to traditional passive teaching. Furthermore, instructors’ personal experiences as students heavily influences their teaching approach ( Gilakjani, 2012). As one of our educators suggested, instructors may need to first experience well-executed blended learning to draw inspiration to design one themselves. One solution could be remote participation in other instructors’ classes to experience a well-executed session. The existing literature supports our conclusion that institutions must support educators in developing effective skills and curricula during an age of rapid change in educational technology ( Daniel *et al.*, 2021; Stojan *et al.*, 2022; Veasuvalingam & Goodson, 2020).

Our multi-institutional study addresses questions educators have posed since the beginning of the COVID-19 pandemic. With the increase in remote learning, instructors have noted a need for best practices based on theory ( Daniel *et al.*, 2021; Gordon *et al.*, 2020). This study provides insights into how emerging technology and teaching methods can align with an existing theoretical framework ( Daniel *et al.*, 2021; Gordon *et al.*, 2020). Our adaptation of an existing curriculum framework could be used as a foundation for developing such “best practices”. In addition, our study provides perspectives from 10 institutions and highlights the potential for cross-institutional communication and collaboration. Whereas one systematic review on undergraduate medical education’s response to COVID-19 noted that most educators tend “to face inward” and multi-institutional studies are rare, our research focuses on how one set of materials can be adapted to fit the goals of many institutions, if instructors apply the concepts of the curriculum framework ( Stojan *et al.*, 2022).

Our study has limitations. First, our study population is limited to educators who inquired about using the M&I videos. We did not systematically find all educators who used the videos after discovering them online in the public domain. Second, we did not collect course evaluation data from our participants. However, our study’s aim was to capture the educators’ experience and not to evaluate course success. To help guide instructors in the post pandemic environment, we focused solely on the educators creating the teaching, not the recipients of the teaching. It may be valuable in future studies to see if students and instructors perceive the education goal of online videos in the same way.

In our study, we found successful integration of external videos into a health professions course is associated with educators who view curriculum design as inter-related elements. When these elements are consistent, educators perceive that their integration of online videos into their curriculum was successful. Consistency between curricular elements seemed to be associated with less institutional pressure on the instructor to teach content instead of concepts. It is important for educators to articulate their aspirations for their students deliberately, so that these goals become the foundation for other curricular elements. Our study also highlights the need to offer dedicated time to developing experienced and enthusiastic educators who will lead successful blended learning courses with effective online video integration.

## Data availability

The data supporting this study is stored on a secure server and only accessible by the research team as described in the data handling requirements approved by the Stanford University Institutional Review Board. The data are not publicly available due to their containing information that could compromise the privacy of research participants. De-identified data are available on request from the corresponding author, KZA, if for the purposes of further research. In the request, please include the reason for requesting data, plans for analysis, and contact information.
