# Supplementary material for: Approaches to integrating online videos into health professions curricula: educators’ perspectives from multiple institutions
Source: MedEdPublish (2016). 2022 Sep 29;12:52. Originally published 2022 Jul 12. [Version 2] doi: 10.12688/mep.19179.2 (PMC10425914; doi:10.12688/mep.19179.2)
Supplement: Peer review history

# Peer review: Reviewer response for version 2

**DOI:** 10.21956/mep.20672.r32617

**Published:** 2022-10-05

**Authors:** Barbara Jennings

**Affiliations:** 1 Norwich Medical School, University of East Anglia, Norwich, UK

The authors have responded thoroughly to the reviewer comments and documented the amendments in their responses to reviewers. Thank you very much, I enjoyed reading this article again about how and why educators use short open-source animated videos in their lessons. The article (with direct links to the open-source resources) will be of great interest to colleagues who curate and use online learning objects within their curriculum, and to others who are focused on principles of instructional design.

Have any limitations of the research been acknowledged?

Yes

Is the work clearly and accurately presented and does it cite the current literature?

Yes

If applicable, is the statistical analysis and its interpretation appropriate?

Not applicable

Are all the source data underlying the results available to ensure full reproducibility?

Yes

Is the study design appropriate and is the work technically sound?

Yes

Are the conclusions drawn adequately supported by the results?

Yes

Are sufficient details of methods and analysis provided to allow replication by others?

Partly

Reviewer Expertise:

Technology enhanced learning and faculty development.

I confirm that I have read this submission and believe that I have an appropriate level of expertise to confirm that it is of an acceptable scientific standard.

# Peer review: Reviewer response for version 2

**DOI:** 10.21956/mep.20672.r32616

**Published:** 2022-10-04

**Authors:** Kenneth Masters

**Affiliations:** 1 Medical Education and Informatics Department, College of Medicine and Health Sciences, Sultan Qaboos University, Muscat, Oman

The authors have addressed all of my concerns, and I am happy to approve this version of the paper.

Have any limitations of the research been acknowledged?

Yes

Is the work clearly and accurately presented and does it cite the current literature?

Yes

If applicable, is the statistical analysis and its interpretation appropriate?

Not applicable

Are all the source data underlying the results available to ensure full reproducibility?

No source data required

Is the study design appropriate and is the work technically sound?

Yes

Are the conclusions drawn adequately supported by the results?

Partly

Are sufficient details of methods and analysis provided to allow replication by others?

Yes

Reviewer Expertise:

Online teaching and learning.

I confirm that I have read this submission and believe that I have an appropriate level of expertise to confirm that it is of an acceptable scientific standard.

# Peer review: Reviewer response for version 1

**DOI:** 10.21956/mep.20546.r32348

**Published:** 2022-07-26

**Authors:** Barbara Jennings

**Affiliations:** 1 Norwich Medical School, University of East Anglia, Norwich, UK

The authors present an interesting qualitative study about the way educators used short open-source animated videos which had been described in a previous evaluation article (Adam *et al.*, 2017 - cited in the introduction of this manuscript; and the videos can be found here). There were some unique and important aspects to this analysis – for example, the focus was on what the educators' intentions and actions were. In addition, the themes that emerged were analysed with respect to an established framework for an integrated health professions curriculum (Lee *et al.*, 2013 – cited in the article).

 The introduction, methods, results, and discussion sections are written clearly and are complemented by a useful table and illustration of the thematic analysis. In the introduction, the authors explore the impact of the pandemic on practice for online learning (with useful citations including a BEME guide) – the focus on video resources that can be used for active learning within the curriculum and (intentional) instructional design principles make this a good read for anyone interested in asynchronous online teaching.

 In the methods section, the recruitment and (semi-structured) interviewing of 10 participants (who lead microbiology or immunology curricula in US medical schools) are described along with the thematic analysis. However, more detail in the methods and results sections would allow a more thorough understanding of the study and its findings.

 Please could the authors consider the following queries about the Methods section:

1. How did the authors select the ten study participants? Could you elaborate on this in the methods section: *“We selected participants from educators who requested access to our open-source M&I videos and formally agreed to be contacted in the future. We emailed the educators directly to ask about participation in our study.”*?
2. Could you link the interview documentation to the manuscript? For example, this could be the list of open-ended questions or the interview guide that was piloted and refined.

Please could the authors consider the following queries about the Results section:

1. In the data analysis section, the authors state *“The key themes were reviewed and refined until team consensus was reached.”* Could this have been explained in more detail in the themes section of the Results?

Ideally, the full data set or metadata would be shared, as described in this F1000 guide, but I note (in the data availability section) that this would not fit with the data handling requirements approved by the Institutional Review Board, and that anonymised data is available on request.

Have any limitations of the research been acknowledged?

Yes

Is the work clearly and accurately presented and does it cite the current literature?

Yes

If applicable, is the statistical analysis and its interpretation appropriate?

Not applicable

Are all the source data underlying the results available to ensure full reproducibility?

Yes

Is the study design appropriate and is the work technically sound?

Yes

Are the conclusions drawn adequately supported by the results?

Yes

Are sufficient details of methods and analysis provided to allow replication by others?

Partly

Reviewer Expertise:

Technology enhanced learning and faculty development.

I confirm that I have read this submission and believe that I have an appropriate level of expertise to confirm that it is of an acceptable scientific standard, however I have significant reservations, as outlined above.

# Response

**Published:** 2022-08-23

**Authors:** Kelly Aluri

**Affiliations:** Stanford University School of Medicine, USA

Thank you for reviewing our paper and for your detailed, constructive feedback. We clarified the following information in our Methods and Results sections:

1. We added some clarifying information about our participant recruitment process: “We recruited participants from educators who requested access to our open-source M&I videos and formally agreed to be contacted in the future. We emailed all educators who requested access to the videos directly to ask about participation in our study. Ten educators agreed to participate in the study. We verified that these ten participants were instructors or course directors of a microbiology and/or immunology course at an accredited U.S. medical or osteopathic school.”
2. We added the interview guide in Appendix 1 to provide more context on our semi-structured interview questions. While not all questions were asked and some new questions arose during each interview, this provided a way for each interview to address our core research questions. 
3. To clarify our key themes in the discussion we clarified:
  “We organized the key themes discussed in this section around a conceptual framework for curriculum development (Lee et al. 2013).”

# Peer review: Reviewer response for version 1

**DOI:** 10.21956/mep.20546.r32342

**Published:** 2022-07-20

**Authors:** Kenneth Masters

**Affiliations:** 1 Medical Education and Informatics Department, College of Medicine and Health Sciences, Sultan Qaboos University, Muscat, Oman

The paper describes a qualitative study regarding the use of videos in microbiology and immunology classes given during the pandemic.

 Overall, the study has been well-conducted and the results well-presented. There are a few issues that do need to be addressed, but I think that these will be done relatively easily.

- “ *To extract lessons to inform the post-pandemic medical education environment, we evaluated how educators from different institutions used an identical set of online medical education videos*.” - This sentence comes too early, as the Introduction is still discussing the background, and this sentence is already describing what the researchers did. In fact, that sentence and quite a bit of the information in the Introduction needs to go into the Methods, especially material from the last paragraph of the Introduction. In addition, if copyright permits, it would be a good idea to replicate the model from Lee *et al*., otherwise readers have to go to that paper to familiarise themselves with it. (The description of the model given in this paper is really not enough. I realise that the authors have provided their Figure 1, but because it does not match the original model exactly, a view of the original model will be useful for the reader).
- A link to the video site would be useful (and also good advertising for the site :-).
- “ *The Institutional Review Board at Stanford University approved our study*” - It is generally considered standard practice to supply the reference number, so, please do so here.
- “ *While Nvivo is a proprietary software, the same analysis can be conducted using open source software such as FreeQDA and QDA Miner Lite*.” - I think it would be best to remove this sentence. Firstly, it is not necessary, and, secondly, as much as I like using QDA Miner Lite, there are some things that it cannot do (e.g. add new data iteratively).
- It would also aid the readers if the authors could have a separate Conclusion (as they have in the Abstract).
- The final paragraph/Conclusion also ignores an important overall finding. Although time is an issue (as the authors have correctly identified), the problem appears to be broader than that and is related to overall institutional support. The first two quotations given in Dimension 4 are crucial: (1) having institutions/faculty acknowledge that maybe, people in other institutions are better-resourced to create better-quality materials, and (2) balancing editing against original creation.  So, the Conclusion does need to identify that overall institution support (including swallowing one’s pride) is required.

**Minor**:

- “ *in 9 different states*” - Generally, numbers less than 10, when used in a sentence, are written out in words.

So, overall, a good and useful study and paper, with a small amount of work required. If the issues are addressed, I will happily change my rating to “Approved”.

Have any limitations of the research been acknowledged?

Yes

Is the work clearly and accurately presented and does it cite the current literature?

Yes

If applicable, is the statistical analysis and its interpretation appropriate?

Not applicable

Are all the source data underlying the results available to ensure full reproducibility?

No source data required

Is the study design appropriate and is the work technically sound?

Yes

Are the conclusions drawn adequately supported by the results?

Partly

Are sufficient details of methods and analysis provided to allow replication by others?

Yes

Reviewer Expertise:

Online teaching and learning.

I confirm that I have read this submission and believe that I have an appropriate level of expertise to confirm that it is of an acceptable scientific standard, however I have significant reservations, as outlined above.

# Response

**Published:** 2022-08-23

**Authors:** Kelly Aluri

**Affiliations:** Stanford University School of Medicine, USA

Thank you for reviewing our paper and for your detailed, constructive feedback. The following changes were made in response to your comments:

1. We moved some of the information from the introduction to the methods to provide more background in the introduction: “To extract lessons to inform the post-pandemic medical education environment, we evaluated how educators from different institutions used an identical set of online medical education videos.”
2. We added a link to the YouTube channel that currently houses the videos.
3. We agree that seeing Lee et al.’s original figure would provide the most context but we will not directly use the image for copyright reasons. We provided more explanation of their original figure in the Methods.
4. The IRB protocol number was added to the Methods.
5. We removed the statement about NVivo alternative softwares.
6. We made our final paragraph a conclusion section, and emphasized the finding that institutions should support curricular development by supporting the use of outside resources and allocating time for training educators.
